# Genome-Wide Association Study Reveals Genetic Loci Associated with Body Measurement Traits in Yanqi Horses

**DOI:** 10.3390/ani16111597

**Published:** 2026-05-24

**Authors:** Weijun Sun, Zhehong Shen, Daoerji Cairen, Penghui Luo, Xinkui Yao, Jun Meng, Yaqi Zeng

**Affiliations:** 1 College of Animal Science, Xinjiang Agricultural University, Urumqi 830052, China; 15352590926@163.com (W.S.); 18858657690@163.com (Z.S.);; 2 Bazhou Animal Husbandry Workstation, Urumqi 841000, China; 3 Animal Husbandry Station of Xinjiang Uygur Autonomous Region, Urumqi 830004, China; 4 Xinjiang Key Laboratory of Horse Breeding and Exercise Physiology, Urumqi 830052, China

**Keywords:** body measurement traits, candidate genes, genome-wide association study, single-nucleotide polymorphism, Yanqi horse

## Abstract

Body measurement traits are a direct production indicator reflecting growth status and guiding genetic selection. Identifying molecular markers associated with body measurement traits could accelerate animal breeding programs. The Yanqi horse, an important indigenous breed in Xinjiang, is primarily distributed in the Bayingolin Mongol Autonomous Prefecture. However, molecular markers linked to body measurement traits in Yanqi horses remain uncharacterized. In the present study, whole-genome resequencing was performed on 183 Yanqi horses, yielding 13,366,672 single-nucleotide polymorphisms (SNPs) after quality control. A genome-wide association study on withers height, body length, heart girth, and cannon bone circumference was conducted using a mixed linear model implemented in GEMMA, with population structure and relatedness controlled using principal components and a genomic kinship matrix. Bonferroni-adjusted thresholds (*p* < 1 × 10^−7^ for significant associations; *p* < 1 × 10^−6^ for suggestive associations) were applied to minimize false positives. A total of 185 single-nucleotide polymorphisms were significantly associated with body measurement traits and 359 candidate genes were annotated within 200 kb upstream and downstream of the significant loci. Among these, five genes, *GABRB1*, *FIGN*, *GABRA4*, *ENSECAG00000051747*, and *COX7B2*, may be implicated in the growth and development of Yanqi horses. Gene Ontology and Kyoto Encyclopedia of Genes and Genomes pathway analyses indicated that these genes are primarily involved in cytoskeletal structures within muscle cells, regulation of the actin cytoskeleton, and neuroactive ligand–receptor interaction pathways. In summary, this study presents novel markers and candidate gene sets associated with body measurement traits in Yanqi horses, offering valuable insights for functional gene investigations and presenting substantial potential for accelerating the breeding of Yanqi horses.

## Simple Summary

While genome-wide association studies (GWASs) have identified body measurement traits loci in commercial horse breeds (e.g., Thoroughbreds, Warmbloods), the genetic architecture of growth traits in endangered Chinese indigenous horses remains largely unexplored. This study addresses this gap by conducting the first GWAS on four body conformation traits (withers height, body length, heart girth, cannon bone circumference) in 183 Yanqi horses, a breed adapted to the high-altitude grasslands of Xinjiang. Unlike previous equine GWASs that focused on racing performance or single-trait analysis in commercial populations, our multi-trait approach identified 185 significant SNPs and 359 candidate genes, with five genes (*GABRB1*, *FIGN*, *GABRA4*, *ENSECAG00000051747*, *COX7B2*) showing pleiotropic associations across all traits. Notably, the top associated regions on ECA1, ECA3, and ECA18 do not overlap with known body measurement trait loci (*LCORL*/*NCAPG*, *HMGA2*) identified in European breeds, suggesting distinct genetic mechanisms underlying body conformation in this indigenous population. These findings provide preliminary evidence for breed-specific genomic markers that may inform conservation breeding strategies for this endangered breed.

## 1. Introduction

Over the past decade, numerous genome-wide association studies (GWASs) have been performed in livestock species, including investigations of various traits in Simmental [1], black goats [2], Cervus nippon [3], Holstein cattle [4], Qinchuan black pigs [5], Tibetan northwest white cashmere goats [6], Hu sheep [7], and Dezhou donkeys [8], among others. Whole-genome sequencing has been employed to characterize population structures and to detect polymorphisms potentially associated with economically significant traits in both livestock and poultry. While equine GWASs have been conducted in commercial or performance-oriented breeds, high-resolution genomic studies of body size traits in indigenous Chinese horse populations remain limited.

In horses, GWASs have primarily focused on racing performance and conformation traits in commercial breeds. For instance, Ropka-Molik et al. [9] identified loci associated with racing performance in 610 Arabian horses using the Equine SNP70 BeadChip. Signer-Hasler et al. [10] conducted a GWAS of body measurement traits (withers height, cannon bone circumference) in 1045 Franches-Montagnes horses, revealing significant associations on ECA3 near the *LCORL*/*NCAPG* locus. Metzger et al. [11] reported that *LCORL* variants on ECA3 are associated with withers height in 214 Warmblood horses, while *HMGA2* on ECA6 has been linked to size variation in ponies [12]. More recently, Staiger et al. [13] identified *ZFAT* on ECA9 as associated with body measurement traits in a multi-breed cohort of 1020 horses. However, these studies predominantly utilized commercial breeds (Thoroughbreds, Arabians, Warmbloods) and focused on performance or conformation traits, with limited attention to indigenous Chinese horse populations and their locally adaptive body measurement traits characteristics.

The Yanqi horse, an indigenous breed adapted to the high-altitude grasslands of Xinjiang, China, represents an important genetic resource with unique physiological characteristics [14]. Body measurement traits in this breed are critical for production performance and draft ability, yet their genomic basis remains unexplored, constraining conservation breeding efforts [15].

Accordingly, this study was designed to identify single-nucleotide polymorphisms (SNPs) [16] significantly associated with four body measurement traits [withers height (WH), body length (BL), heart girth (HG), and cannon bone circumference (CBC)] in Yanqi horses through GWAS [9] methodologies. Withers height reflects skeletal growth and overall body frame; body length relates to body conformation and growth development; heart girth indicates thoracic capacity, body mass, and muscular development; and cannon bone circumference reflects limb robustness and load-bearing capacity. These traits are directly linked to breeding objectives for production performance, draft ability, and local adaptation in Yanqi horse conservation programs [10], while other morphometric parameters such as body weight, body diagonal length, and rump height were not included. Simultaneously, candidate genes and biological pathways potentially influencing these traits were screened based on the corresponding SNP loci. We hypothesized that specific genomic regions are significantly associated with body size traits in Yanqi horses and that these regions may contain candidate genes involved in skeletal growth, muscle development, or body conformation. These findings provide molecular markers valuable for the selective breeding of Yanqi horses.

## 2. Materials and Methods

### 2.1. Experimental Animals and Tissue Sample Collection

A total of 183 healthy Yanqi horses were randomly selected as experimental subjects, comprising 36 stallions and 147 mares, aged between 2 months (0 years) and 13 years. All experimental animals originated from a single population maintained at the Baoqi Yanqi Horse Conservation Farm in Bayinbuluke Town, Hejing County, Bayingolin Mongol Autonomous Prefecture. These animals were raised under broadly similar management conditions, including standardized feeding regimens and housing practices, thereby minimizing environmental heterogeneity. Blood samples (10 mL) were drawn from the jugular vein using disposable vacuum blood collection tubes containing ethylenediaminetetraacetic acid as an anticoagulant. After collection, the samples were immediately mixed thoroughly with the anticoagulant, aliquoted, and stored in liquid nitrogen tanks prior to transport to the laboratory for DNA extraction.

### 2.2. Phenotype Data Sources and Measurement Methods

Body measurement traits, including WH, BL, HG, and CBC, were measured on the same group of Yanqi horses by a single individual using a measuring tape and measuring stick. Each trait was measured three times, and the average value was calculated. Descriptive statistical analyses of the phenotypic data were performed using IBM SPSS Statistics 27 software.

### 2.3. DNA Quality Control

Genomic DNA was isolated from the collected blood samples using the standard phenol–chloroform extraction method. The concentration of the extracted DNA was quantified using a NanoDrop2000 (Thermo Fisher Scientific, Waltham, MA, USA) ultra-micro spectrophotometer, while its integrity was evaluated through agarose gel electrophoresis. All DNA samples were preserved at −80 °C.

### 2.4. Whole-Genome Resequencing

Qualified genomic DNA samples were transported on dry ice to Tianjin Kangpusen Agricultural Technology Co., Ltd. (Tianjin, China). for whole-genome sequencing. A library with an insert size of 350 bp was prepared, and paired-end sequencing was performed with a read length of 150 bp using the DNBSEQT7 sequencing platform (MGI Tech Co., Ltd., Tianjin, China).

### 2.5. Sequencing Read Quality Control

To ensure the reliability of downstream information analysis, fastp (v 0.23.4) software (https://github.com/OpenGene/fastp, accessed on 3 February 2026) was employed to conduct rigorous quality control processing on the raw data files (FASTQ format) generated by paired-end sequencing. The parameters fastp -i -I -o -O -w 4 -q 20 -n 2 -u 30 were configured to remove low-quality reads and adapter sequences, thereby producing clean reads and providing a foundation for subsequent data analysis. The criteria applied for data quality control were as follows:

(1) Reads containing adapter sequences were discarded;
(2) Paired reads were removed if the proportion of ambiguous bases (N) in a read exceeded 2% of the total base count;
(3) Paired reads were discarded if the proportion of low-quality bases (Q ≤ 20) in a read exceeded 30% of the total bases.

### 2.6. Alignment to Reference Genome

After quality control of sequencing data, the BWA MEM algorithm in BWA ((v 0.7.17)) software (https://github.com/lh3/bwa, accessed on 23 February 2026) was employed to align the high-quality clean reads (FASTQ format) to the horse reference genome (EquCab 3.0). The alignment parameters were configured as -t 4 -M -R ‘@RG\tID:$i\tLB:$i\tPL\tSM:$i’, and BAM format files containing the alignment results were generated. Subsequently, the SortSam and MarkDuplicates modules of PICARD (v 3.4.0) software (https://broadinstitute.github.io/picard/, accessed on 27 February 2026) were utilized to sort the unsorted BAM files by genomic coordinates by specifying the SORT_ORDER=coordinate parameter, while the REMOVE_DUPLICATES=true parameter was applied to eliminate duplicate reads, thereby yielding high-quality reads (BAM format). The bamqc module of QualiMap was then used to perform quality assessment on the processed BAM files. Finally, the BaseRecalibrator and ApplyBQSR modules of Genome Analysis Toolkit (GATK) (Cambridge, MA, USA) were executed to carry out base quality score recalibration, thereby enhancing the accuracy of sequencing data and ensuring reliable variant calling for subsequent genetic analyses.

### 2.7. Variant Calling and Quality Control

Raw SNPs were identified and genotyped using the HaplotypeCaller, GenotypeGVCFs, and SelectVariants modules of GATK. Initially, variant information was called from BAM files through the HaplotypeCaller module, producing GVCF (Genome VCF) format files to store the variant data. To ensure the accuracy of variant calling, the minimum mapping quality threshold was set to --minimum-mapping-quality 30. Subsequently, the GenomicsDBImport tool in GATK was executed to import multiple GVCF files into a GenomicsDB database, thereby enabling centralized storage and facilitating subsequent processing via the GenotypeGVCFs module. Parameters were configured as --intervals 1 --reader-threads 1 --batch-size 50 to enhance the efficiency of joint genotyping. Genotypes were then generated from the GVCF files using the GenotypeGVCFs module with EquCab 3.0 as the reference genome, and the parameter max-alternate-alleles 1 was specified to limit the number of alternate alleles to one. Finally, the SelectVariants module was employed to extract and filter variant sites from the VCF files, resulting in raw VCF files (SNPs). Quality control was conducted using the VariantFiltration module, in accordance with GATK-recommended hard filtering criteria, ultimately yielding clean SNPs. To improve the quality of variant detection, all raw SNPs were filtered using the “Variant Filtration” module in GATK with the following parameters: QD < 2.0, FS > 60.0, SOR > 3.0, MQ < 40.0, MQRankSum < −12.5, QUAL < 30.0, and ReadPosRankSum < −8.0. These filtering criteria follow the GATK Best Practices Workflow recommendations to ensure high-confidence variant calls.

### 2.8. Variant Annotation

Functional annotation of clean SNPs was carried out using the table_annovar.pl module of ANNOVAR (version 2020-06-08) software, based on the horse reference genome annotation file (https://ftp.ensembl.org/pub/release-113/gtf/equus_caballus/Equus_caballus.EquCab3.0.113.gtf.gz, accessed on 5 March 2026). During the annotation process, the parameter -buildver EquCab3.0 was specified to define the genome build version, while -protocol refGene and -operation g were selected to designate the annotation protocol. This approach enabled the precise determination of whether variants were located within genes and identified the specific genes in which the variants were present.

### 2.9. SNP-Level Quality Control

Raw data were subjected to effective loci screening using PLINK (v 1.9) software [17], based on the following filtering criteria: a minor allele frequency (MAF) greater than 5%, an individual call rate lower than 90%, and an SNP missing rate lower than 10%. The parameters were configured as “--maf 0.05 --mind 0.1 --geno 0.1 --chr-set 31”. Given the sample size of 183 animals, a MAF threshold of 0.05 was chosen because rare variants are difficult to test reliably in modest sample sizes and may introduce instability into association estimates [18]. The missingness thresholds (individual call rate < 90% and SNP missing rate < 10%) were applied to remove low-confidence markers and individuals while retaining sufficient genomic coverage for downstream analysis.

### 2.10. GWAS

GWAS analysis was performed using the mixed linear model (MLM) [19] implemented in GEMMA (v 0.98.5) software [20]. Sex, age, farm effect, and the first three principal components derived from PCA were included as fixed-effect covariates in the model. The MLM equation is as follows:

*Y* = *SNP* + *PCs* + *kinship* + *e*

where Y denotes the phenotypic vector, SNP represents the fixed effect vector, PCs indicate the principal components employed to correct for population structure, Kinship denotes the kinship matrix, and e refers to the random residual effect vector. The K matrix represents kinship relationships derived from SNP markers.

To minimize the inflated false positive rate introduced by multiple testing, a multiple testing correction was applied to the GWAS results. A Bonferroni-adjusted significance threshold of 1 × 10^−7^ and a suggestive threshold of 1 × 10^−6^ were adopted. Finally, Manhattan and quantile–quantile (Q–Q) plots were generated using the CMplot package in R for visualization of GWAS outcomes.

### 2.11. Candidate Gene and Functional Enrichment Analysis

Based on the EquCab 3.0 horse reference genome in Ensembl, the 200 kb regions upstream and downstream of significant loci were annotated using ANNOVAR software [21]. The resulting candidate genes were annotated and subsequently subjected to Gene Ontology (GO) enrichment analysis via the DAVID platform (https://fsabcl-david01p.ncifcrf.gov/tools.jsp, accessed on 5 March 2026) [22].

### 2.12. Supplementary Data Description

Supplementary Materials supporting this study include Supplementary Table S1, containing sample-level sequencing quality control metrics for all 183 individuals; Supplementary Tables S2–S5, providing complete GWAS results for withers height, body length, heart girth, and cannon bone circumference, respectively, including chromosome, genomic position, SNP ID, *p*-values, and nearest gene annotation for all variants exceeding the suggestive significance threshold (*p* < 1 × 10^−6^).

## 3. Results

### 3.1. Descriptive Statistics of Body Measurement Traits in Yanqi Horses

The coefficients of variation (CV%) for the body measurement traits of 183 Yanqi horses were all below 1.5% (Table 1), suggesting a high degree of uniformity within this closed conservation population. As noted above, this low variation is characteristic of genetically homogeneous populations with limited gene flow and standardized management practices. While such reduced phenotypic variance may constrain the total heritable variance available for GWAS detection, it also reflects the controlled environmental conditions at the conservation farm, which minimize non-genetic sources of variation and may enhance the signal-to-noise ratio for genetic associations. Among the four traits, HG exhibited the greatest variation (CV% = 1.13%), likely reflecting individual differences in muscle development that are less constrained by genetic homogeneity than skeletal dimensions.

### 3.2. Genomic Data Statistics

**Figure 1:**
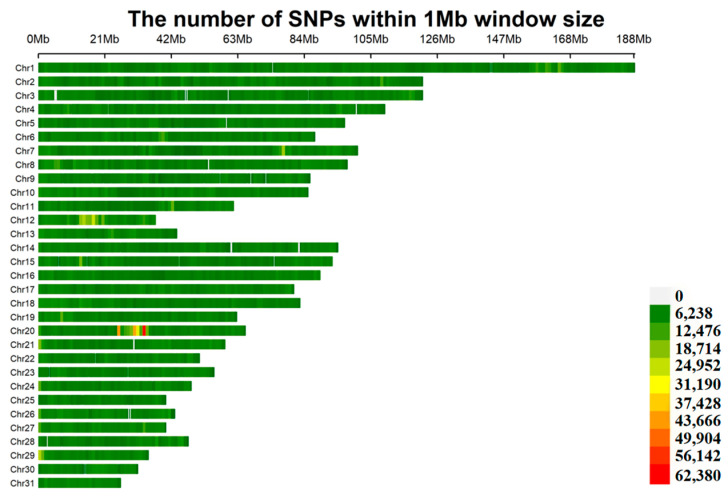
Distribution of SNP loci on autosomes in Yanqi horses.

**Figure 2:**
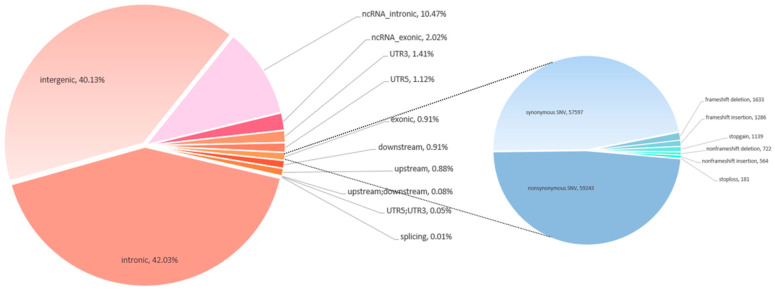
Functional annotation of variant loci in Yanqi horses.

A total of 35,721,465,026 clean reads were generated in this study, with an average mapping rate of 99.84% and an average sequencing depth of 11.39×. Detailed information is presented in Supplementary Table S1. Through sequencing, a total of 27,310,794 SNPs were identified, with an overall genotyping rate of 99.92%, indicating exceptionally high coverage. Following quality control, 13,366,672 SNPs were retained and found to be evenly distributed across the 31 autosomes of Yanqi horses, rendering them suitable for subsequent GWAS analysis (Figure 1). SNPs located within gene regions were found in intronic and exonic regions, accounting for 5,616,877 and 122,236 SNPs, respectively, corresponding to 42.03% and 0.91% of the total SNPs. Within the exonic regions, 59,243 non-synonymous and 57,597 synonymous mutations were identified (Figure 2).

### 3.3. GWAS of WH Traits in Yanqi Horses

**Figure 3:**
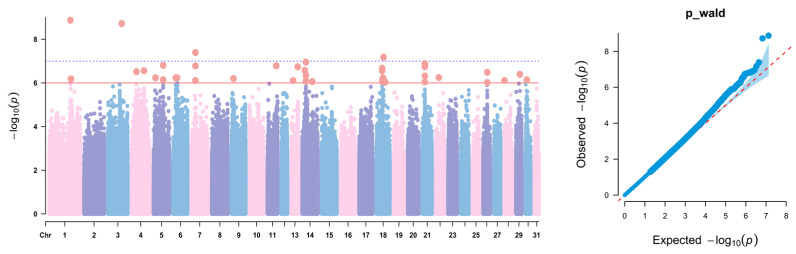
A Manhattan plot and quantile–quantile plot for the withers height trait in Yanqi horses. Pink/blue alternating blocks represent different chromosomes (Chr 1–31); pink dots indicate significant SNP loci exceeding the threshold.

The GWAS results indicated that 45 SNPs fell within the suggestive significance threshold (*p* < 1 × 10^−6^), co-localized with 79 candidate genes. Among these, six SNP loci exhibited significant associations (*p* < 1 × 10^−7^), with notable peaks observed on chromosomes Chr1, Chr3, Chr7, Chr14, Chr18, and Chr21 (Figure 3). The Q–Q plot showed minimal deviation from the expected null distribution at low *p*-values, with a genomic inflation factor λ = 1.01, indicating that the MLM with PCs and the kinship matrix adequately controlled for population stratification (Figure 3). These 45 significant SNPs were further analyzed and annotated using ANNOVAR software within a 200 kb window, both upstream and downstream of the SNP loci significantly associated with WH. The most significant SNP was located at position 128,108.446 kb on chromosome 1, with a *p*-value of 1.34 × 10^−9^, and was annotated to seven genes: *HACD3*, *DENND4A*, *DPP8*, *IGDCC3*, *IGDCC4*, *INTS14*, and *SLC24A1*. Additionally, another significantly associated SNP on the same chromosome was mapped to the *VPS13C* gene. Annotations for the remaining significant loci are provided in Supplementary Table S2.

### 3.4. GWAS of BL Traits in Yanqi Horses

**Figure 4:**
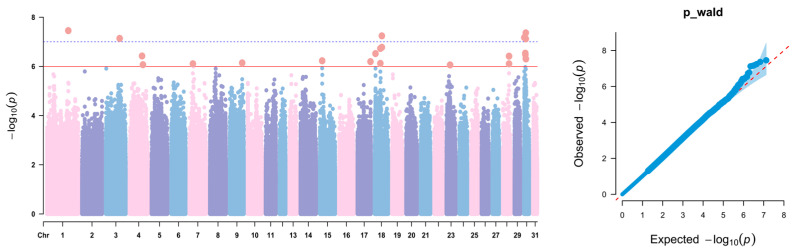
A Manhattan plot and quantile–quantile plot for the body length trait in Yanqi horses. Pink/blue alternating blocks represent different chromosomes (Chr 1–31); pink dots indicate significant SNP loci exceeding the threshold.

The GWAS results identified a total of 25 SNPs within the suggestive significance threshold (*p* < 1 × 10^−6^), co-localized with 79 candidate genes. Among these, six SNP loci exhibited significant associations (*p* < 1 × 10^−7^), with prominent signals detected on chromosomes Chr1, Chr3, Chr18, and Chr30 (Figure 4). The Q–Q plot showed close adherence to the expected null distribution, with λ = 0.99, indicating negligible inflation (Figure 4). For comparative analysis and gene annotation, the 25 significantly associated SNPs were examined using ANNOVAR software within a 200 kb window upstream and downstream of the BL-associated loci. The most significant SNP was located at position 128,108.446 kb on chromosome 1, with a *p*-value of 3.51 × 10^−8^, and was annotated to seven genes: *HACD3*, *DENND4A*, *DPP8*, *IGDCC3*, *IGDCC4*, *INTS14*, and *SLC24A1*. Annotations for the remaining significant loci are listed in Supplementary Table S3.

### 3.5. GWAS of HG Traits in Yanqi Horses

**Figure 5:**
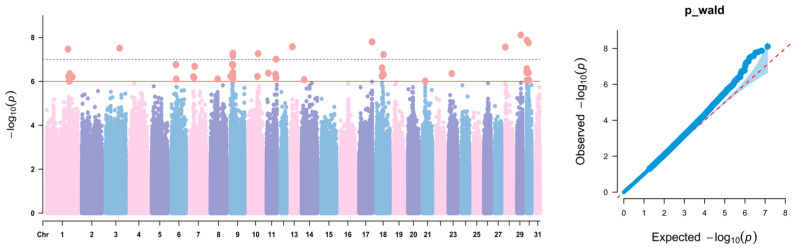
A Manhattan plot and quantile–quantile plot for the heart girth trait in Yanqi horses. Pink/blue alternating blocks represent different chromosomes (Chr 1–31); pink dots indicate significant SNP loci exceeding the threshold.

The GWAS results identified a total of 51 SNPs within the suggestive significance threshold (*p* < 1 × 10^−6^), co-localized with 109 candidate genes. Among these, 13 SNP loci exhibited significant associations (*p* < 1 × 10^−7^), with elevated signals observed on chromosomes Chr1, Chr3, Chr9, Chr10, Chr11, Chr13, Chr17, Chr18, Chr28, Chr29, and Chr30 (Figure 5). The Q–Q plot exhibited slight deviation at the tail, with λ = 1.03, suggesting modest inflation potentially attributable to the higher polygenic variance of heart girth; however, this value remains within the acceptable range (λ < 1.05) for well-controlled GWASs (Figure 5). These 51 significant SNPs were subjected to comparative analysis and gene annotation using ANNOVAR software, based on a 200 kb region upstream and downstream of each HG-associated SNP locus. The most significant locus was located at position 23,147.732 kb on chromosome 29, with a *p*-value of 7.81 × 10^−9^, and was annotated to the calcium/calmodulin-dependent protein kinase ID gene, *CAMK1D*. Annotations for the remaining significant loci are provided in Supplementary Table S4.

### 3.6. GWAS of CBC Traits in Yanqi Horses

**Figure 6:**
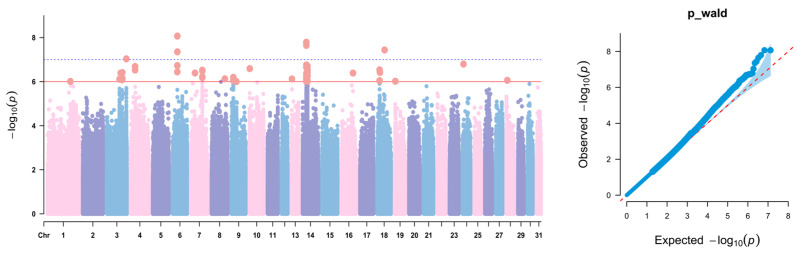
A Manhattan plot and quantile–quantile plot for the cannon bone circumference trait in Yanqi horses. Pink/blue alternating blocks represent different chromosomes (Chr 1–31); pink dots indicate significant SNP loci exceeding the threshold.

The GWAS results identified a total of 64 SNPs within the suggestive significance threshold (*p* < 1 × 10^−6^), co-localized with 92 candidate genes. Among these, seven SNP loci exhibited significant associations (*p* < 1 × 10^−7^), with prominent signals observed on chromosomes Chr3, Chr6, Chr14, and Chr18 (Figure 6). The Q–Q plot showed excellent fit to the null distribution, with λ = 0.98, indicating no evidence of inflation (Figure 6). These 64 significant SNPs were subjected to comparative analysis and gene annotation using ANNOVAR software, based on a 200 kb region both upstream and downstream of each CBC-associated SNP locus. The most significant locus was located at position 28,069.889 kb on chromosome 6, with a *p*-value of 8.53 × 10^−9^, and was annotated to four genes: *A0A5F5PYI1_HORSE, PEX26, TUBA8,* and *USP18*. Additionally, seven other significantly associated SNP loci mapped to the same chromosome were also annotated to the same four genes. Annotations for the remaining significant loci are listed in Supplementary Table S5.

### 3.7. Candidate Gene Venn Diagram of Yanqi Horse

**Figure 7:**
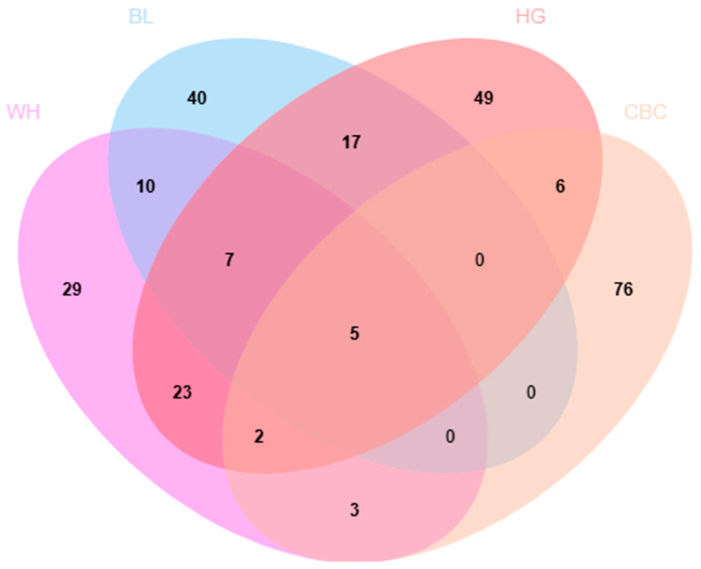
Venn diagram of candidate genes in Yanqi horses.

As shown in Figure 7, the candidate genes shared by four body measurement traits of Yanqi horse are *GABRB1, FIGN, GABRA4, ENSECAG00000051747*, and *COX7B2.*

### 3.8. Functional Annotation and Enrichment Analysis of Candidate Genes for WH Traits in Yanqi Horses

**Figure 8:**
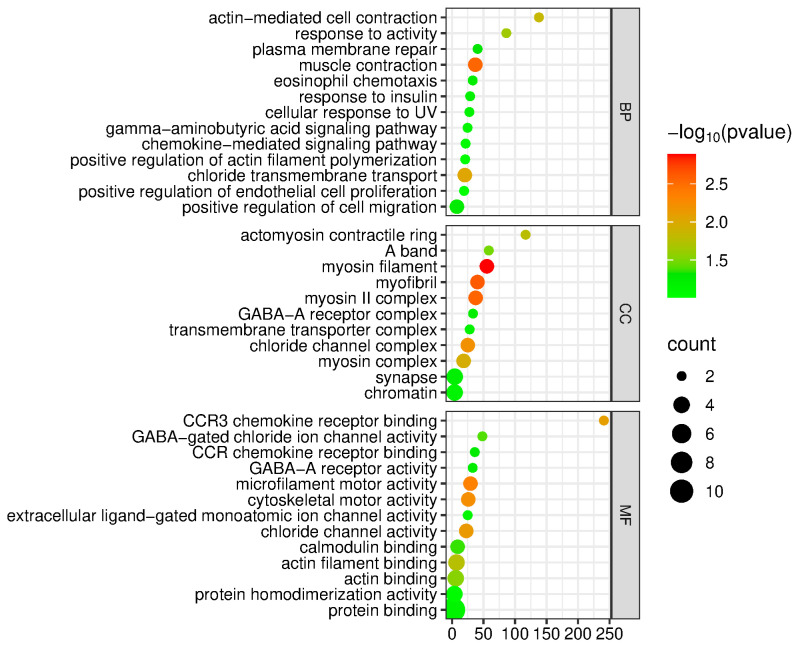
GO bubble enrichment plot of candidate genes for the withers height trait in Yanqi horses.

To further investigate the biological roles of candidate genes significantly associated with WH traits in Yanqi horses, GO functional annotation was conducted. The annotation framework was categorized into three domains: biological process, cellular component, and molecular function. As illustrated in Figure 8 and ranked by *p*-value, the enriched functional categories within each domain were as follows: (1) Biological process: muscle contraction, chloride transmembrane transport, actin-mediated cell contraction, etc. (2) Cellular component: myosin filament, myofibril, myosin II complex, etc. (3) Molecular function: microfilament motor activity, cytoskeletal motor activity, chloride channel activity, etc.

**Figure 9:**
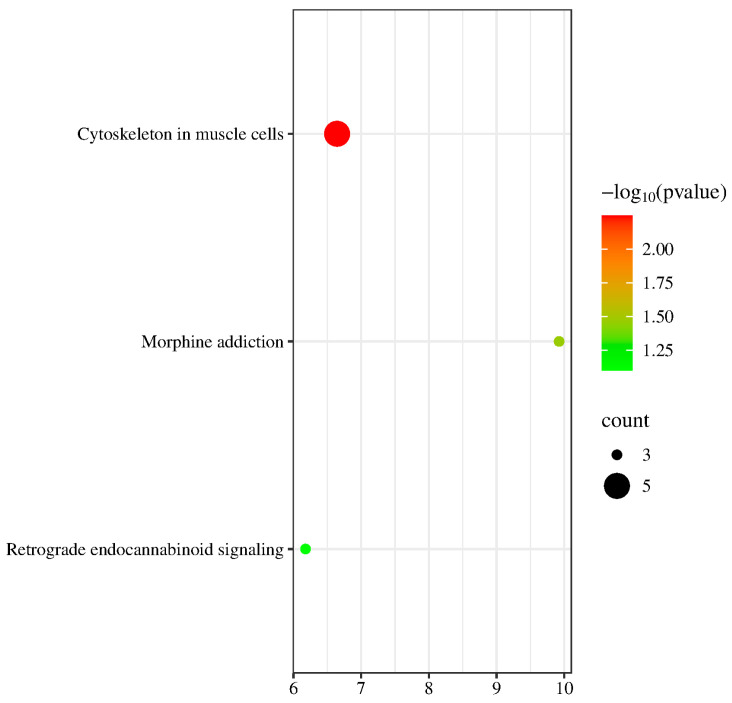
KEGG bubble enrichment plot of candidate genes for WH traits in Yanqi horses.

To elucidate the metabolic pathways involving these candidate genes in vivo, Kyoto Encyclopedia of Genes and Genomes (KEGG) pathway enrichment analysis was performed on the gene cluster significantly associated with WH in Yanqi horses. A bubble enrichment plot was generated based on statistically significant pathways, as shown in Figure 9. These genes were found to be significantly enriched in three primary pathways: cytoskeleton in muscle cells, and retrograde endocannabinoid signaling. Among these, the highest number of genes was enriched in the cytoskeleton in muscle cells pathway.

### 3.9. Functional Annotation and Enrichment Analysis of Candidate Genes for BL Traits in Yanqi Horses

**Figure 10:**
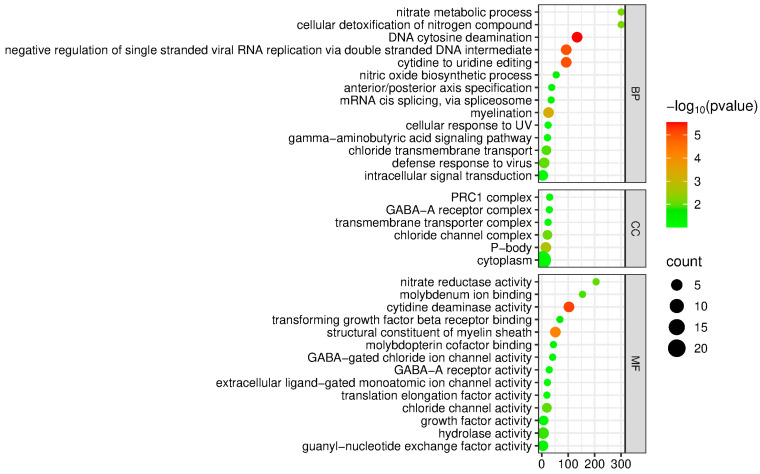
GO bubble enrichment plot of candidate genes for body length in Yanqi horses.

To further investigate the functions of candidate genes significantly associated with BL traits in Yanqi horses, GO functional annotation was conducted. The annotation structure was categorized into three domains: biological process, cellular component, and molecular function. As illustrated in Figure 10 and ranked by *p*-value, the enriched GO categories within each domain were as follows: (1) Biological process: DNA cytosine deamination, cytidine to uridine editing, negative regulation of single-stranded virus via double-stranded DNA intermediate, etc. (2) Cellular component: P-body, chloride channel complex, PRC1 complex, etc. (3) Molecular function: cytidine deaminase activity, structural constituent of myelin sheath, nitrate reductase activity, etc.

**Figure 11:**
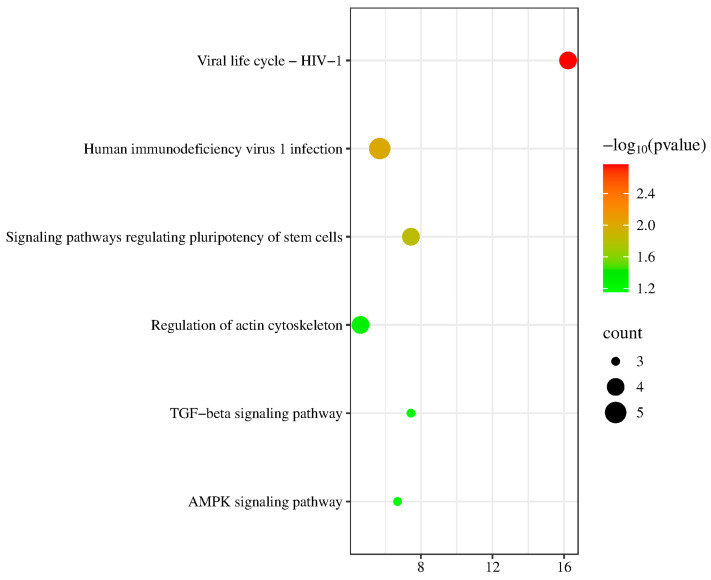
KEGG bubble enrichment plot of candidate genes for body length traits in Yanqi horses.

To elucidate the metabolic pathways in which the candidate genes participate in vivo, KEGG pathway enrichment analysis was conducted on the gene cluster significantly associated with BL in Yanqi horses. A bubble enrichment plot was generated based on statistically significant pathways, as illustrated in Figure 11. These candidate genes were significantly enriched in six pathways: TGF-beta signaling pathway, signaling pathways regulating pluripotency of stem cells, AMPK signaling pathway, human immunodeficiency virus 1 infection, and regulation of actin cytoskeleton. Among these, the highest number of genes was enriched in the human immunodeficiency virus 1 infection pathway.

### 3.10. Functional Annotation and Enrichment Analysis of Candidate Genes for HG Traits in Yanqi Horses

**Figure 12:**
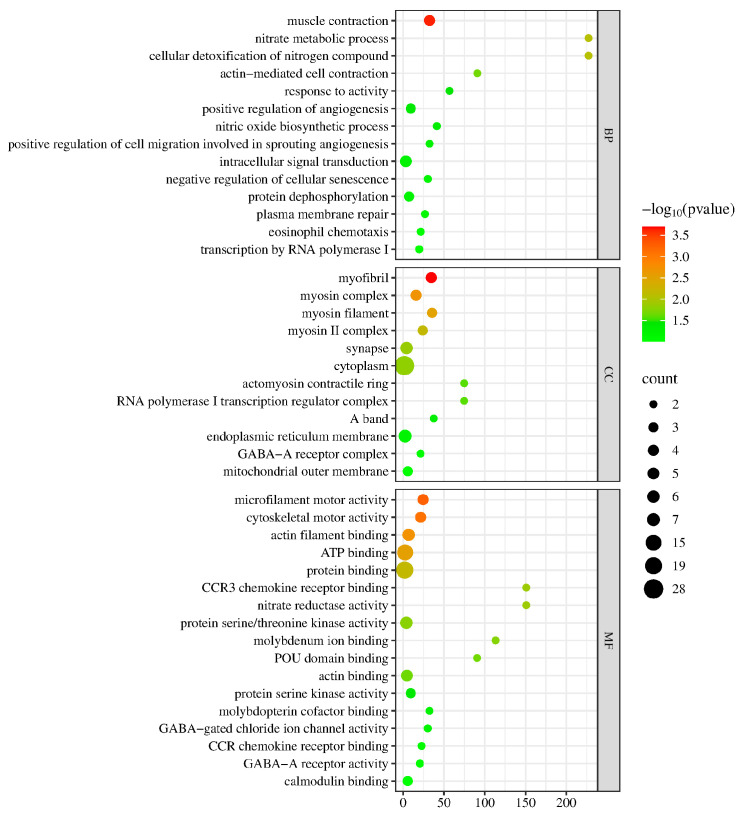
GO bubble enrichment plot of candidate genes for heart girth trait in Yanqi horses.

To further investigate the biological functions of candidate genes significantly associated with HG traits in Yanqi horses, GO functional annotation was conducted. The annotation structure was classified into three domains: biological process, cellular component, and molecular function. As illustrated in Figure 12 and ranked by *p*-value, the enriched GO categories within each domain were as follows: (1) Biological process: muscle contraction, nitrate metabolic process, cellular detoxification of nitrogen compound, etc. (2) Cellular component: myofibril, myosin complex, myosin filament, etc. (3) Molecular function: microfilament motor activity, cytoskeletal motor activity, etc.

**Figure 13:**
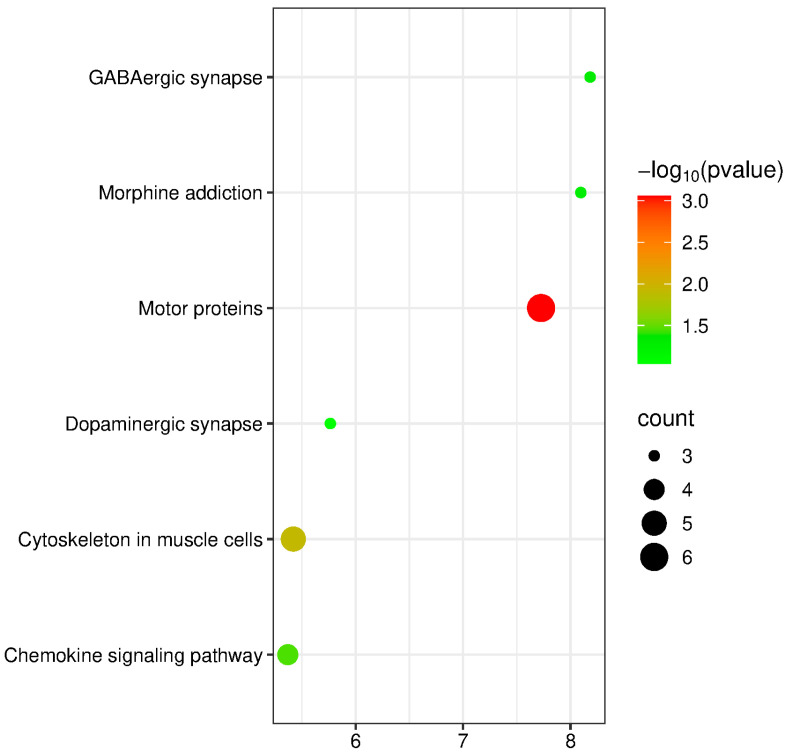
KEGG bubble enrichment plot of candidate genes for heart girth trait in Yanqi horses.

To elucidate the metabolic pathways in which these candidate genes participate in vivo, KEGG pathway enrichment analysis was conducted on the gene cluster significantly associated with HG in Yanqi horses. A bubble enrichment plot was generated based on statistically significant pathways, as shown in Figure 13. These genes were significantly enriched in six pathways: GABAergic synapse, morphine addiction, motor proteins, dopaminergic synapse, cytoskeleton in muscle cells, and chemokine signaling pathway. Among these, the highest number of genes was enriched in the motor protein pathway.

### 3.11. Functional Annotation and Enrichment Analysis of Candidate Genes for CBC Traits in Yanqi Horses

**Figure 14:**
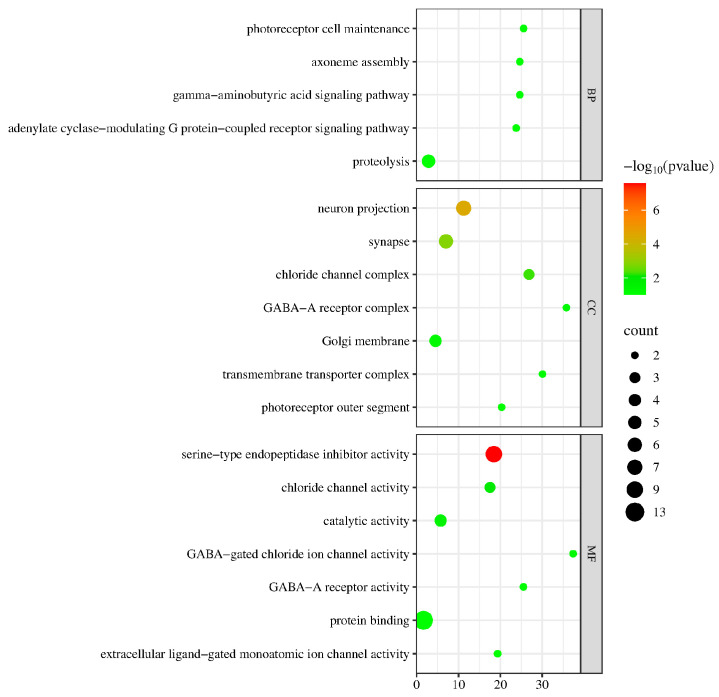
GO bubble enrichment plot of candidate genes for cannon bone circumference in Yanqi horses.

To further investigate the biological functions of candidate genes significantly associated with CBC traits in Yanqi horses, GO functional annotation was carried out. The annotation structure was organized into three categories: biological process, cellular component, and molecular function. As illustrated in Figure 14 and ranked by *p*-value, the enriched GO terms in each category were as follows: (1) Biological process: photoreceptor cell maintenance, gamma-aminobutyric acid signaling pathway, axoneme assembly, etc. (2) Cellular component: neuron projection, synapse, chloride channel complex, etc. (3) Molecular function: serine-type endopeptidase inhibitor activity, chloride channel activity, catalytic activity, etc.

**Figure 15:**
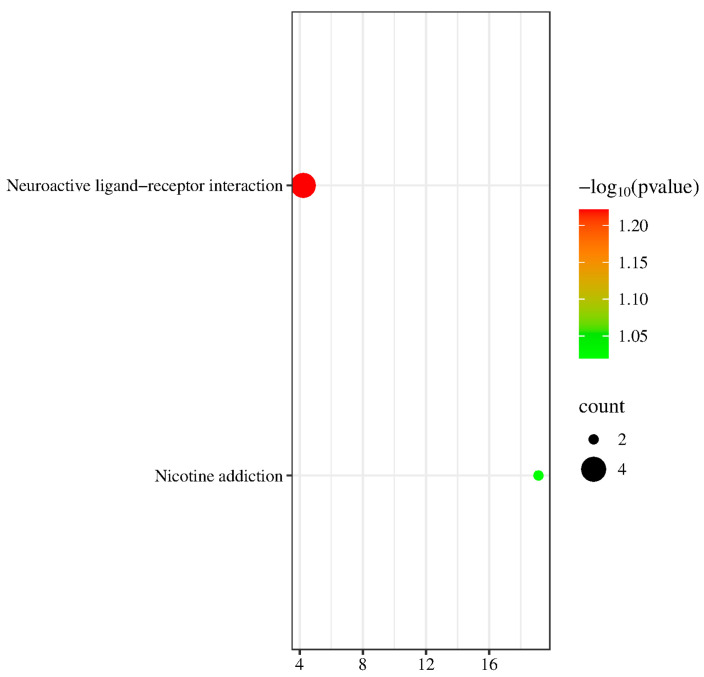
KEGG bubble enrichment plot of candidate genes for cannon bone circumference traits in Yanqi horses.

To elucidate the metabolic pathways in which candidate genes participate in vivo, KEGG pathway enrichment analysis was conducted on the gene cluster significantly associated with CBC in Yanqi horses. A bubble enrichment plot was generated based on statistically significant pathways, as illustrated in Figure 15. These candidate genes were significantly enriched in two pathways: nicotine addiction and neuroactive ligand–receptor interaction. Among these, the highest number of genes was enriched in the neuroactive ligand–receptor interaction pathway.

## 4. Discussion

### 4.1. Overview of GWAS Findings

In this study, a GWAS was conducted on four body measurement traits in 183 Yanqi horses using the MLM based on resequencing data; given the limited sample size, this GWAS is underpowered for detecting small-effect variants and should be interpreted as an exploratory marker discovery study. Following Bonferroni multiple testing correction of the *p*-values obtained, a total of 185 loci were identified as significantly associated with body measurement traits in Yanqi horses. Following Bonferroni multiple testing correction of the *p*-values obtained, a total of 185 loci were identified as significantly associated with body measurement traits in Yanqi horses. Gene annotation was performed within a 200 kb region, both upstream and downstream of these significant loci, leading to the identification of 359 candidate genes. Among these, 45 loci were significantly associated with WH, 25 with BL, 51 with HG, and 64 with CBC. Notably, five overlapping candidate genes, *GABRB1*, *FIGN*, *GABRA4*, *ENSECAG00000051747*, and *COX7B2*, were annotated across all four traits and are known to be involved in multiple biological processes, with previous studies reporting their association with body measurement traits development.

Comparison with established equine body size loci reveals notable divergence. While *LCORL/NCAPG* (ECA3) and *HMGA2* (ECA6) represent the most robustly replicated loci in European commercial breeds [10,11,12], and *ZFAT* (ECA9) was recently identified in a multi-breed cohort [13], the top associated regions in our Yanqi horse study (ECA1, ECA3 at ~128.1 Mb, and ECA18) do not overlap with these established loci. Specifically, no significant signals were detected at the *LCORL/NCAPG* (~105.5 Mb), *HMGA2* (81.6 Mb), or *ZFAT* (29.4 Mb) positions, suggesting that body size regulation in this indigenous Chinese breed may involve distinct genetic mechanisms.

### 4.2. Model Adequacy and Population Control

The genomic inflation factors (λ) for the four traits ranged from 0.98 to 1.03 (WH: 1.01; BL: 0.99; HG: 1.03; CBC: 0.98), all within the acceptable range for well-controlled GWASs (λ < 1.05) [23]. These values indicate that the mixed linear model, incorporating both principal components and a genomic kinship matrix, successfully accounted for population structure and cryptic relatedness. The Q–Q plots showed minimal systematic deviation from the expected null distribution, with distinct separation of tail signals only at the extreme *p*-value range, consistent with true polygenic associations rather than inflationary artifacts. Notably, the slightly higher λ for HG (1.03) may reflect greater polygenic architecture of heart girth compared to skeletal traits (WH, BL, CBC), though this difference is marginal and does not compromise the validity of the associations.

### 4.3. Candidate Genes: From Genotype to Phenotype

Interpreting genotype to phenotype: The gamma-aminobutyric acid (GABA) receptor gene *GABRB1* may indirectly regulate growth hormone (GH) secretion through the hypothalamic–pituitary–growth axis, thereby modulating skeletal development and body measurement traits in animals. In a genome-wide association analysis of Dutch Holstein cattle, Harmen P. Doekes [24] et al. reported that genetic markers proximal to *GABRB1* were significantly associated with body measurement traits characteristics such as withers height and hip width. Similarly, Wossenie Mebratie [25] et al. identified SNPs near *GABRB1* in a broiler chicken GWAS, which were associated with tibial length and body weight, and hypothesized that these variants might affect body measurement traits through GABA signaling pathways regulating skeletal development and the growth axis. Both *GABRA4* and *GABRB1*, members of the GABA receptor gene family, are essential in the nervous system and function by mediating GABAergic inhibitory neurotransmission [26]. In a bovine genome-wide study, Muhammad S. Tahir [27] et al. suggested that GABA signaling transmitted via *GABRA4* receptors acts in coordination with N-methyl-D-aspartate and α-amino-3-hydroxy-5-methyl-4-isoxazolepropionic acid receptors, leading to calcium ion channel activation, elevated intracellular calcium concentrations, and subsequent stimulation of gonadotropin-releasing hormone secretion, ultimately impacting pubertal and body measurement traits development. Furthermore, Zhao et al. [28] demonstrated in Simmental cattle that the GABA synaptic pathway was related to live weight and contributed to the regulation of feeding behavior. In horses, GABAergic signaling in the hypothalamus regulates growth hormone-releasing hormone (GHRH) pulsatility, which directly impacts skeletal growth rates. The Yanqi horse, adapted to high-altitude grasslands with seasonal nutritional fluctuations, may exhibit unique GABA receptor variants that modulate growth plasticity in response to environmental constraints. Thus, *GABRA4* and *GABRB1* may influence body measurement traits in Yanqi horses predominantly through mechanisms involving neural (GABA receptor neurotransmission) and hormonal (growth hormone-releasing hormone secretion) regulation, which warrants further empirical validation. *FIGN* encodes an ATP-dependent microtubule-severing enzyme of the AAA-ATPase family, which plays a fundamental role in cytoskeletal dynamics, mitosis, and ciliary function. It is hypothesized that *FIGN* may affect body measurement traits by modulating skeletal growth, muscle development, and fat metabolism. G. A. Cox [29] et al. demonstrated that *FIGN* influences mammalian development, with its mutant form, fidgetin, promoting osteocyte differentiation, thus implicating it as a potential regulator of skeletal and muscular growth. Therefore, it is proposed that *FIGN* may influence body measurement traits development in Yanqi horses through metabolic mechanisms involving skeletal and adipose tissue regulation, a hypothesis that requires further investigation. *COX7B2*, an auxiliary subunit of cytochrome c oxidase in the mitochondrial electron transport chain, is involved in oxidative phosphorylation and ATP synthesis [30]. Sumona Akter [31] et al. demonstrated that *COX7B2* plays a role in the assembly of mitochondrially encoded core subunits of cytochrome c oxidase, and that defects in this assembly process lead to mitochondrial dysfunction, substantially reducing exercise capacity and subsequently impairing growth and development in animals. As a positional candidate gene identified in the present GWAS, *COX7B2* has known relevance to mitochondrial function and energy metabolism. Its possible relationship with growth or body size traits in Yanqi horses, whether through effects on energy production efficiency or other mechanisms, remains to be determined and requires functional validation. *ENSECAG00000051747* currently lacks experimentally validated functional annotation, and its evidence as a candidate gene for body measurement traits is primarily based on statistical association and bioinformatic prediction. The specific regulatory mechanisms of this gene remain to be further elucidated through experimental approaches such as RNA-seq, RACE cloning, or CRISPR-based perturbation.

It is important to emphasize that the genes identified in this study represent candidate associations derived from statistical genomic screening, and that cross-species evidence cited herein is indirect. Definitive causal relationships between these variants and body measurement traits in Yanqi horses require functional validation through expression analysis (e.g., RNA-seq of growth plate cartilage or skeletal muscle), eQTL mapping, or gene editing models. The current study provides a foundational resource for such downstream investigations.

### 4.4. Pathway Enrichment and Biological Interpretation

At the pathway level, GO analysis revealed that, at the biological process level, the candidate genes were significantly enriched in signal transduction, organic substance response, and specific catalytic activity processes. Analysis of cellular components indicated that these genes were predominantly localized within myosin complexes, myofibrils, and synaptic structures. Molecular function enrichment further identified ion channel activity and membrane protein functionality as prominent characteristics. KEGG pathway analysis demonstrated that significantly enriched signaling pathways were chiefly concentrated in the cytoskeleton in muscle cells, regulation of actin cytoskeleton, and neuroactive ligand-receptor interaction. A growing body of research has highlighted the essential involvement of the cytoskeletal system in regulating muscle development and body measurement traits. Petra Gimpel et al. [32] demonstrated that mutations in the cytoskeletal protein Nesprin-1 result in aberrant nuclear positioning within muscle cells, ultimately leading to the manifestation of muscular dystrophy symptoms. This finding elucidated that cytoskeletal proteins contribute not only to cellular architecture maintenance but also directly influence normal muscle development. Complementary findings by Jianyan Zeng et al. [33] indicated that cytoskeletal pathways in muscle cells are engaged in critical biological processes such as myotube fusion and myofiber organization, primarily by regulating actin polymerization and microtubule dynamics, thereby impacting skeletal muscle morphology and physiological function. Mutations in Nesprin-1 may contribute to abnormalities in muscle cell positioning and potentially hinder body measurement traits development in Yanqi horses. At the molecular level, Thomas D. Pollard et al. [34] demonstrated that within the actin cytoskeleton regulatory pathway, filamentous actin provides structural support and mechanical propulsion to cells, thereby contributing to cellular shape and motility, which in turn influence organismal growth and phenotypic size variation. Furthermore, Sarah J. Heasman et al. [35] revealed that regulation of the actin cytoskeleton via Rho GTPase signaling modulates cell proliferation and migration, consequently impacting muscle tissue development and expansion. These collective observations highlight the close association between myofibril organization and muscle mass, as well as body measurement traits. Alterations in myofibril architecture may modulate muscle cell morphology and dynamics, leading to differences in body measurement traits among Yanqi horses. Beyond the cytoskeletal network, endocrine signaling pathways have also been implicated in the regulation of body measurement traits. Andrea M. Hanson et al. [36] reported that the hypothalamic-pituitary-liver axis orchestrates postnatal growth and development through the GHRH-GH-IGF-1 signaling cascade. F. Lupu et al. [37] demonstrated that GH and IGF-1 act both independently and synergistically to drive postnatal growth, with these pathways serving as key regulators of phenotypic body measurement traits differentiation. Thyroid hormone (TH) also plays a pivotal role in skeletal development and linear growth. J. H. Duncan Bassett et al. [38] revealed that TH deficiency halts skeletal maturation and growth, and that neuroactive ligand-receptor interaction pathways influence TH secretion, thereby modulating body measurement traits. Therefore, TH may influence skeletal development and growth in Yanqi horses, and TH insufficiency may contribute to delayed skeletal maturation, ultimately resulting in observable differences in body measurement traits.

## 5. Conclusions

In this study, we performed a whole-genome resequencing-based GWAS of four body measurement traits in Yanqi horses and identified candidate genomic regions associated with withers height, body length, heart girth, and cannon bone circumference. The results provide preliminary evidence that body conformation in this indigenous horse population may involve genomic regions related to cytoskeletal organization, muscle cell structure, neuroactive ligand–receptor signaling, and growth-related biological processes. However, because of the limited sample size, single-population design, broad age range, moderate sequencing depth, and lack of independent validation, these findings should be interpreted as candidate associations rather than confirmed causal loci. Future studies should validate lead SNPs in larger and independent Yanqi horse populations and integrate gene expression, eQTL, and functional genomic analyses to clarify the biological roles of prioritized candidate genes. Overall, this study provides a useful genomic resource for Yanqi horses and lays a preliminary foundation for future marker-assisted selection and conservation breeding in this indigenous breed.

## Figures and Tables

**Table 1:** Descriptive statistics of body measurement traits.

Traits	Number	Mean	SD	Max	Min	CV%
Withers height (cm)	183	132.07	0.96	152.00	98.00	0.73
Body length (cm)	183	131.28	1.30	160.00	90.00	0.99
Heart girth (cm)	183	150.01	1.70	180.00	90.00	1.13
Cannon bone circumference (cm)	183	16.64	0.13	20.00	12.00	0.78

## Data Availability

The data and materials used in this research are available from the corresponding author on request.

## Supplementary Materials

The following supporting information can be downloaded at: https://www.mdpi.com/article/10.3390/ani16111597/s1, Table S1: Statistics of Yanqi horse sequence comparison results; Table S2: GWAS analysis results of withers height traits in Yanqi horses; Table S3: GWAS analysis results of body length traits in Yanqi horses; Table S4: GWAS analysis results of heart girth traits in Yanqi horses; Table S5: GWAS analysis results of cannon bone circumference traits in Yanqi horses.
