# Supplementary material for: Genome-Wide Association Study Reveals Genetic Loci Associated with Body Measurement Traits in Yanqi Horses
Source: Animals (Basel). 2026 May 24;16(11):1597. doi: 10.3390/ani16111597 (PMC13255931; doi:10.3390/ani16111597)
Supplement: Supplementary file 1 [file animals-16-01597-s001.zip › animals-4283704-supplementary.pdf]

Supplementary Material

1. SUPPLEMENTARY TABLES

Supplementary Table S1. Statistics of Yanqi horse sequence comparison results

Horse number	Number of reads	Number of mapped reads	GC Percentage	Mean coverage Data
23C147F	175215013	175,092,324 (99.93%)	42.79%	10.1,799X
23C148	187057167	186,861,879 (99.9%)	42.64%	10.1,601X
23C149	186047160	185,931,230 (99.94%)	42.99%	10.1,828X
23C150	170614845	170,402,516 (99.88%)	42.06%	9.666X
23C151	173786930	173,611,211 (99.9%)	43.05%	10.1,665X
23C152	185113532	184,972,535 (99.92%)	42.78%	10.719X
23C153	192496724	192,296,985 (99.9%)	42.52%	11.1,206X
23C154	173910238	173,783,204 (99.93%)	42.69%	10.025X
23C155	215106447	214,962,979 (99.93%)	42.65%	12.1,225X
23C156	218924551	218,784,179 (99.94%)	42.54%	12.1,532X
23C158	187616039	187,501,920 (99.94%)	42.76%	10.1,632X
23C159	200379415	200,251,456 (99.94%)	43.39%	11.628X
23C160	224826423	224,681,883 (99.94%)	42.79%	13.1,619X
23C161	179067443	178,947,281 (99.93%)	42.63%	10.1,187X
23C162	178035286	177,911,430 (99.93%)	42.54%	10.1,942X
23C163	210175767	210,031,616 (99.93%)	42.49%	12.1,121X
23C164	216127765	215,989,218 (99.94%)	42.77%	12.1,327X

23C165	215475779	215,332,550 (99.93%)	42.79%	12.1,677X
23C166	220923933	220,775,224 (99.93%)	42.84%	12.1,016X
23C167	229951306	229,808,754 (99.94%)	43.16%	13.1,385X
23C168	225259814	225,099,755 (99.93%)	43.36%	13.1,518X
23C169	183415616	183,299,745 (99.94%)	43.20%	10.1,021X
23C170	220767552	220,613,404 (99.93%)	42.77%	12.1,188X
23C171	185176626	185,008,633 (99.91%)	43.41%	10.1,325X
23C172	201224203	201,089,487 (99.93%)	43.02%	11.1,066X
23C173	226436222	226,292,736 (99.94%)	43.01%	13.1,662X
23C174	194725752	194,600,035 (99.94%)	45.28%	11.1,342X
23C175	218940022	218,739,132 (99.91%)	43.46%	12.1,339X
23C176	221253162	221,118,027 (99.94%)	43.03%	12.1,607X
23C177	179428774	179,311,881 (99.93%)	43.10%	10.1,218X
23C178	240273492	240,129,869 (99.94%)	43.34%	13.1,729X
23C179	176053208	175,936,052 (99.93%)	43.11%	10.1,178X
23C180	237647462	237,493,474 (99.94%)	42.76%	13.1,122X
23C181	187216551	187,114,123 (99.95%)	43.05%	10.1,325X
23C182	186104213	185,941,248 (99.91%)	43.75%	10.1,682X
23C183	202011471	201,881,281 (99.94%)	43.23%	11.1,182X
23C184	208860088	208,718,811 (99.93%)	43.42%	12.185X
23C185	224875212	224,726,178 (99.93%)	43.53%	13.1,583X
23C186	202506517	202,316,858 (99.91%)	44.40%	11.1,022X

23C187	220122943	219,985,728 (99.94%)	43.85%	12.1,161X
23C188	168094832	167,962,304 (99.92%)	43.41%	9.1,375X
23C189	212674460	212,478,587 (99.91%)	43.52%	12.1,949X
23C190	219422023	219,264,409 (99.93%)	43.08%	12.805X
23C191	168642277	168,523,749 (99.93%)	42.81%	9.1,863X
23C192	252940603	252,780,984 (99.94%)	43.37%	14.1,912X
23C193	210779432	210,637,801 (99.93%)	43.35%	12.1,243X
23C194	219141006	218,986,563 (99.93%)	43.11%	12.1,147X
23C195	195087458	194,874,644 (99.89%)	43.38%	11.1,856X
23C196	165294541	165,132,986 (99.9%)	44.18%	9.1,537X
23C197	243151026	242,977,410 (99.93%)	43.50%	14.1,843X
23C198	239476394	239,316,783 (99.93%)	43.34%	13.1,516X
23C199	165739929	165,605,654 (99.92%)	43.18%	9.1,945X
23C200	174448664	174,301,992 (99.92%)	43.07%	10.1,909X
23C201	201658492	201,530,414 (99.94%)	43.72%	11.1,713X
23C202	185350841	185,225,989 (99.93%)	43.59%	10.1,404X
23C203	205384769	205,248,691 (99.93%)	43.54%	12.1,349X
23C204	184791931	184,649,233 (99.92%)	43.39%	10.1,115X
23C205	214509947	214,349,698 (99.93%)	44.22%	12.1,753X
23C206	253275807	253,103,305 (99.93%)	43.82%	14.1,645X
23C207	197880267	197,741,137 (99.93%)	43.36%	11.1,514X
23C208	224702599	224,546,065 (99.93%)	43.52%	13.132X

23C209	171965533	171,852,874 (99.93%)	44.04%	10.1,353X
23C210	221032655	220,889,822 (99.94%)	44.72%	12.896X
23C211	211930847	211,779,717 (99.93%)	43.45%	12.1,818X
23C212	187068817	186,892,071 (99.91%)	44.19%	10.1,339X
23C214	235893153	235,725,723 (99.93%)	43.51%	13.1,414X
23C215	178005634	177,889,171 (99.93%)	43.27%	10.1,075X
23C216	206897403	206,744,578 (99.93%)	43.47%	12.1,929X
23C217	211503715	211,296,457 (99.9%)	43.48%	12.1,706X
23C218	224286469	224,135,488 (99.93%)	43.67%	13.1,198X
23C219	159712863	141,775,283 (88.77%)	43.89%	8.1,397X
23C220	198152090	197,970,610 (99.91%)	44.26%	11.1,947X
23C221	193113323	192,987,507 (99.93%)	44.33%	11.1,982X
23C222	275723821	275,561,628 (99.94%)	43.57%	16.1,064X
23C223	194217393	194,094,211 (99.94%)	42.66%	11.1,059X
23C224	201252091	201,061,616 (99.91%)	42.96%	11.1,916X
23C225	214980435	214,832,746 (99.93%)	43.87%	12.1,738X
23C226	222793091	222,614,071 (99.92%)	43.89%	12.1,971X
23C227	169085550	168,940,956 (99.91%)	43.12%	9.1,027X
23C228	203129436	203,017,312 (99.94%)	44.09%	11.1,788X
23C229	170846904	170,644,107 (99.88%)	43.09%	10.1,107X
23C231	168122022	168,010,630 (99.93%)	44.48%	9.1,529X
23C232	186894845	186,778,416 (99.94%)	44.11%	10.1,521X

23C233	211550700	211,404,354 (99.93%)	43.43%	12.1,755X
23C234	236483632	236,318,225 (99.93%)	42.98%	13.1,357X
23C235	172702982	172,490,355 (99.88%)	43.26%	10.1,048X
23C236	223685849	223,521,346 (99.93%)	43.11%	13.1,123X
23C237	187502466	187,316,226 (99.9%)	43.32%	10.1,698X
23C238	185285609	185,103,554 (99.9%)	43.68%	10.1,416X
23C239	187029621	186,897,587 (99.93%)	43.95%	10.1,463X
23C242	175384827	175,214,930 (99.9%)	43.88%	10.1,826X
23C244	200221192	200,024,617 (99.9%)	44.05%	11.1,255X
23C245	217817337	217,729,616 (99.96%)	44.47%	12.1,538X
23C248	183467165	183,278,029 (99.9%)	42.85%	10.1,973X
23C249	196791333	196,653,139 (99.93%)	43.02%	11.498X
23C250	198911392	198,771,970 (99.93%)	43.21%	11.1,258X
23C252	197162838	197,021,578 (99.93%)	43.54%	11.1,403X
23C253	206448146	206,306,550 (99.93%)	43.20%	12.1,224X
23C254	191004443	190,873,029 (99.93%)	43.49%	11.1,055X
23C255	173207040	173,090,144 (99.93%)	43.17%	10.1,555X
23C256	199455593	199,308,865 (99.93%)	43.22%	11.1,833X
23C257	221228452	220,992,071 (99.89%)	42.90%	12.1,929X
23C258	190903326	190,709,348 (99.9%)	42.62%	11.1,158X
23C259	194764524	194,634,463 (99.93%)	42.75%	11.1,299X
23C260	168851012	168,726,652 (99.93%)	42.69%	9.1,619X

23C261	212866325	212,633,122 (99.89%)	43.32%	12.1,383X
23C262	185587103	185,320,077 (99.86%)	43.58%	10.861X
23C263	196622682	196,435,038 (99.9%)	42.87%	11.1,144X
23C264	207078409	204,805,777 (98.9%)	42.91%	11.1,749X
23C265	169219409	169,084,850 (99.92%)	42.63%	9.879X
23C266	170326027	170,139,480 (99.89%)	42.70%	9.1,339X
23C267	175291296	175,168,183 (99.93%)	42.54%	10.265X
23C268	191072893	190,943,291 (99.93%)	43.15%	11.1,727X
23C269	185402419	185,252,920 (99.92%)	42.91%	10.1,625X
23C270	262159022	261,973,533 (99.93%)	43.47%	15.1,003X
23C271	218858556	218,582,140 (99.87%)	43.48%	12.1,801X
23C272	176893678	176,706,689 (99.89%)	43.36%	10.1,558X
23C273	227415448	227,242,943 (99.92%)	42.64%	13.1,263X
23C274	178319169	178,103,604 (99.88%)	42.59%	10.1,396X
23C275	200013874	199,854,216 (99.92%)	42.91%	11.1,455X
23C276	176664228	176,531,789 (99.93%)	43.07%	10.348X
23C277	206786708	206,634,284 (99.93%)	43.71%	12.1,104X
23C278	192128769	189,287,544 (98.52%)	43.15%	11.1,853X
23C279	188233272	188,095,441 (99.93%)	43.93%	11.1,269X
23C280	179867164	179,736,735 (99.93%)	43.13%	10.1,646X
23C281	240180693	240,004,558 (99.93%)	43.32%	14.1,765X
23C282	211743745	211,572,140 (99.92%)	43.67%	12.1,587X

23C283	219478078	219,299,078 (99.92%)	43.98%	12.1,333X
23C284	199871255	199,721,471 (99.93%)	43.60%	11.1,102X
23C285	192883888	192,746,219 (99.93%)	44.48%	11.1,891X
23C286	170693051	170,567,760 (99.93%)	43.62%	10.1,297X
23C287	211116561	210,966,123 (99.93%)	43.93%	12.1,841X
23C288	200917330	200,763,749 (99.92%)	42.42%	11.1,187X
23C289	182437014	182,204,628 (99.87%)	43.20%	10.1,888X
23C290	168626231	168,454,344 (99.9%)	43.48%	9.886X
23C291	175296655	175,154,435 (99.92%)	43.86%	10.1,549X
23C292	168128924	168,005,608 (99.93%)	43.99%	9.1,494X
23C293	166700406	166,526,060 (99.9%)	42.66%	9.1,826X
23C294	195656911	195,496,527 (99.92%)	42.89%	11.1,494X
23C295	161470445	161,316,060 (99.9%)	45.10%	9.1,441X
23C296	212666542	212,502,844 (99.92%)	43.20%	12.1,828X
23C297	168269397	168,155,937 (99.93%)	43.47%	9.1,746X
23C298	186248090	186,078,109 (99.91%)	43.11%	10.1,544X
23C299	226458977	226,295,775 (99.93%)	43.72%	13.1,753X
23C300	178351735	178,215,054 (99.92%)	43.55%	10.1,394X
23C301	179346104	179,225,440 (99.93%)	43.65%	10.1,194X
23C302	168442963	168,272,860 (99.9%)	43.26%	9.1,805X
23C303	217838535	217,670,747 (99.92%)	42.86%	12.1,876X
23C304	186400687	186,274,388 (99.93%)	44.25%	10.1,156X

23C305	176999460	176,882,723 (99.93%)	45.76%	10.1,275X
23C308	176488069	176,370,165 (99.93%)	43.66%	10.1,342X
23C309	178563761	178,361,998 (99.89%)	44.37%	10.1,469X
23C310	164173869	164,026,806 (99.91%)	44.62%	9.1,861X
23C311	199592963	199,432,901 (99.92%)	44%	11.1,832X
23C312	178785361	178,658,666 (99.93%)	44.02%	10.1,356X
23C313	169065525	168,855,718 (99.88%)	43.32%	9.1,688X
23C314	166912911	166,765,200 (99.91%)	44.74%	9.1,939X
23C315	217306640	217,125,314 (99.92%)	43.62%	12.1,088X
23C316	173523258	173,412,554 (99.94%)	44.23%	10.1,309X
23C317	196552679	196,403,949 (99.92%)	44.34%	11.1,042X
23C320	173577075	173,437,736 (99.92%)	43.36%	10.1,232X
23C321	168508985	168,387,256 (99.93%)	44.27%	9.1,506X
23C322	169097443	168,973,506 (99.93%)	43.87%	9.1,966X
23C323	182522231	182,386,775 (99.93%)	43.22%	10.1,921X
23C324	234037822	233,789,369 (99.89%)	44.13%	13.1,945X
23C325	170220088	170,044,730 (99.9%)	43.43%	9.1,865X
23C327	183277512	183,159,020 (99.94%)	43.73%	10.1,135X
23C328	182981060	182,815,561 (99.91%)	42.75%	10.1,359X
23C329	220902127	220,683,261 (99.9%)	43.12%	12.1,637X
23C330	196698405	196,327,842 (99.81%)	43.74%	11.1,196X
23C331	199686499	199,208,759 (99.76%)	44.36%	11.1,653X

23C332	168262207	168,069,512 (99.89%)	44.55%	9.1,225X
23C333	166261621	165,828,768 (99.74%)	45.28%	9.1,828X
23C334	179135359	178,753,596 (99.79%)	44.86%	10.1,397X
23C335	162566785	162,375,387 (99.88%)	46.38%	9.1,678X
23C336	171782004	171,562,866 (99.87%)	45.68%	10.1,308X
23C337	168219594	168,048,613 (99.9%)	45%	9.1,301X
23C338	163109867	162,881,438 (99.86%)	44.07%	9.1,567X
23C339	187109109	186,975,658 (99.93%)	43.59%	10.1,608X
23C340	167549954	167,378,977 (99.9%)	44.02%	9.1,998X
23C341	168495393	168,275,746 (99.87%)	43.50%	9.1,586X
23C342	175450968	175,262,280 (99.89%)	43.75%	10.1,676X
23C343	161952145	161,774,138 (99.89%)	43.65%	9.487X

Supplementary Table S2. GWAS analysis results of withers height traits in Yanqi horses

**Chromosome**	**Base pair**	**P-value**	**Candidate genes**	**Type**
1	128108446	1.31,074E-09	HACD3、DENND4A、DPP8、IGDCC3、IGDCC4、INTS14、SLC24A1	exonic
3	83602269	1.1,006E-09	GABRB1、GABRA4、COX7B2	exonic
7	24226205	4.01,134E-08	CADM1	exonic
18	43907924	6.1,506E-08	FIGN、ENSECAG00000051747	exonic
14	22054210	1.11,274E-07	SGCD、A0A9L0R6T5_HORSE、A0A9L0SHH5_HORSE	exonic
21	15168750	1.21,682E-07	PLK2	exonic
21	15221125	1.31,848E-07	ENSECAG01,001,031,578;ENSECAG00000040189	ncRNA_exonic

21	15178867	1.51,494E-07	ENSECAG01,001,031,578;ENSECAG 00000040189	ncRNA _exonic
5	58320527	1.51,553E-07	ENSECAG01,001,031,831;ENSECAG 00000057959	interge nic
5	58320528	1.51,553E-07	ENSECAG01,001,031,831;ENSECAG 00000057959	interge nic
11	53463664	1.61,689E-07	PIRT、MYH1、MYH2、MYH3、SCO1、 ADPRM、TMEM220	exonic
7	24229556	1.1,556E-07	CADM1	exonic
21	15175153	1.81,094E-07	ENSECAG01,001,031,578;ENSECAG 00000040189	ncRNA _exonic
21	15176784	1.81,094E-07	ENSECAG01,001,031,578;ENSECAG 00000040189	ncRNA _exonic
13	38818375	1.1,137E-07	RBFOX1	exonic
18	35133308	2.11,071E-07	RPRM	exonic
14	15428847	2.61,974E-07	ENSECAG00000028173	ncRNA _exonic
4	74694768	2.71,927E-07	ST7、CFTR、ASZ1、WNT2、 A0A9L0SYG0_HORSE	exonic
18	35159166	2.71,983E-07	RPRM	exonic
4	28689561	3.01,036E-07	SEMA3A	exonic
26	24870779	3.21,232E-07	CYYR1、ADAMTS1	exonic
29	23147732	4.01,079E-07	CAMK1D	exonic
14	21933038	4.41,309E-07	SGCD、A0A9L0SHH5_HORSE	exonic
21	15089872	4.81,898E-07	PLK2、GAPT	exonic
22	17858523	5.61,131E-07	GPCPD1、SHLD1	exonic
5	12218511	5.1,984E-07	ASTN1、BRINP2	exonic
18	39950515	5.81,561E-07	WDSUB1、BAZ2B、TANC1	exonic
6	23642946	5.81,457E-07	RBM44、RAB17、PRLH、LRRFIP1、 COL6A3、MLPH	exonic
6	14751687	5.81,327E-07	ENSECAG01,001,021,392;ENSECAG 01,001,031,608;ENSECAG01,001,041, 022;ENSECAG00000052439	ncRNA _exonic

9	10793203	6.21,098E-07	ZFHX4	exonic
18	43906517	6.41,623E-07	FIGN、ENSECAG00000051747	exonic
1	131519687	6.51,438E-07	VPS13C	exonic
5	58300750	7.01,751E-07	ENSECAG01,001,031,831;ENSECAG00000057959	intergenic
30	7673888	7.1,684E-07	ACBD3、H3-3A、SDE2、LIN9、PARP1、MIXL1	exonic
18	36333154	7.31,343E-07	KCNJ3	exonic
14	22164408	7.61,619E-07	SGCD、A0A9L0R6T5_HORSE、A0A9L0SHH5_HORSE	exonic
7	24230590	7.71,567E-07	CADM1	exonic
28	46311	7.81,261E-07	NONE;ENSECAG00000050704	intergenic
13	11117851	7.81,279E-07	POM121C、POR、RHBDD2、CCL26、HIP1、CCL24、A0A9L0SRJ7_HORSE	exonic
14	22147537	8.61,151E-07	SGCD、A0A9L0R6T5_HORSE、A0A9L0SHH5_HORSE	exonic
14	60453346	8.61,123E-07	SLC25A46、TMEM232	exonic
18	36327659	8.71,328E-07	KCNJ3	exonic
21	15220469	9.1,503E-07	ENSECAG01,001,031,578;ENSECAG00000040189	ncRNA_exonic
18	54950287	9.21,103E-07	MTX2	exonic
26	24824909	9.61,004E-07	CYYR1	exonic

Supplementary Table S3. GWAS analysis results of body length traits in Yanqi horses

**Chromosome**	**Base pair**	**P-value**	**Candidate genes**	**Type**
1	128108446	3.51,987E-08	DPP8、SLC24A1、INTS14、HACD3、DENND4A、IGDCC4、IGDCC3	exonic
30	10546788	4.31,684E-08	AIDA、DISP1、FAM177B、BROX、BPNT1、IARS2	exonic
18	43907924	5.71,285E-08	FIGN、ENSECAG00000051747	exonic

30	1484883	6.61,982E-08	AKT3	exonic
3	83602269	7.21,373E-08	GABRB1、GABRA4、COX7B2	exonic
30	10594573	7.51,259E-08	AIDA、DISP1、FAM177B、BROX、BPNT1、IARS2、RAB3GAP2	exonic
18	43906517	1.1,719E-07	FIGN、ENSECAG00000051747	exonic
18	34649180	1.1,683E-07	A0A9L0S410_HORSE、ARL6IP6、PRPF40A、FMNL2	exonic
30	7673888	2.91,318E-07	ACBD3、H3-3A、SDE2、LIN9、PARP1、MIXL1	exonic
18	5280215	3.01,751E-07	ENSECAG01,001,051,786;ENSECAG00000055907	ncRNA_exonic
30	8085674	3.31,251E-07	PYCR2、TMEM63A、EPHX1、ENAH、LOC101,051,366、LOC101,051,366、SRP9	exonic
4	74787213	3.71,446E-07	CFTR、ASZ1、WNT2、CTTNBP2、A0A9L0SYG0_HORSE	exonic
28	37034075	3.81,241E-07	SUN2、APOBEC3Z2A-Z2、APOBEC3H、CBX7、JOSD1、GTPBP1、CBX6、PDGFB、DNAL4、NPTXR、ENSECAG01,001,041,549、LOC100146802	exonic
28	37034076	3.81,241E-07	SUN2、APOBEC3Z2A-Z2、APOBEC3H、CBX7、JOSD1、GTPBP1、CBX6、PDGFB、DNAL4、NPTXR、ENSECAG01,001,041,549、LOC100146802	exonic

28	37034079	3.81,241E-07	SUN2、APOBEC3Z2A-Z2、APOBEC3H、CBX7、JOSD1、GTPBP1、CBX6、PDGFB、DNAL4、NPTXR、ENSECAG01,001,041,549、LOC100146802	exonic
30	10593438	4.81,325E-07	AIDA、DISP1、FAM177B、RAB3GAP2、BROX、BPNT1、IARS2	exonic
30	11317153	4.91,861E-07	HLX、C30H1orf115、MARK1、MARK1、MTARC2	exonic
15	13654031	5.81,801E-07	A0A5F5PMV9_HORSE、LOC101,781,721、LOC101,621,535、A0A9L0RXL2_HORSE、LOC111,761,183、A0A9L0RFM9_HORSE、	exonic
17	78515477	6.31,662E-07	ARHGEF7、TEX29	exonic
9	74781570	7.11,547E-07	OC90、EFR3A、ENSECAG00000056142	exonic
18	34719923	7.51,676E-07	A0A9L0S410_HORSE、ARL6IP6、PRPF40A、FMNL2	exonic
7	20622120	7.81,236E-07	POU2AF1、PPP2R1B、BTG4、HOATZ、LAYN、SIK2	exonic
28	37034068	7.81,924E-07	SUN2、APOBEC3Z2A-Z2、APOBEC3H、CBX7、JOSD1、GTPBP1、	exonic

4	80194328	8.51,042E-07	CBX6、PDGFB、DNAL4、NPTXR、ENSECAG01,001,041,549、LOC100146802 TMEM229A	exonic
23	22066233	8.61,522E-07	DOCK8、KANK1	exonic

Supplementary Table S4. GWAS analysis results of heart girth traits in Yanqi horses

Chromosome	Base pair	P-value	Candidate genes	Type
29	23147732	7.81,193E-09	CAMK1D	exonic
30	1484883	1.1,432E-08	AKT3	exonic
17	78515477	1.51,801E-08	ARHGEF7、TEX29	exonic
30	11317153	1.71,294E-08	HLX、C30H1orf115、MARK1、MTARC1、MTARC2	exonic
13	11117851	2.61,819E-08	POM121C、POR、RHBDD2、CCL26、HIP1、CCL24、A0A9L0SRJ7_HORSE	exonic
28	46311	2.71,352E-08	ENSECAG00000050704	intergenic
3	83602269	3.01,175E-08	GABRB1、GABRA4、COX7B2	exonic
1	128108446	3.41,181E-08	DPP8、SLC24A1、INTS14、HACD3、DENND4A、IGDCC4、IGDCC3	exonic
9	14133150	5.21,374E-08	KCNB2	exonic
10	56919043	5.41,455E-08	PREP、A0A9L0SXE5_HORSE	exonic
18	43907924	5.91,985E-08	FIGN、ENSECAG00000051747	exonic
9	14125795	6.71,275E-08	KCNB2	exonic
11	57478613	9.81,181E-08	PMP22、TEKT3、CDRT4	exonic
9	10803698	1.71,872E-07	ZFHX4	exonic
6	25950265	1.71,644E-07	ANKMY1、AQP12、GPC1、KIF1A、	exonic

			RNPEPL1、CAPN10、DUSP28、A0A9L0SJG8_HORSE	
9	14142367	1.71,833E-07	KCNB2	exonic
9	14145590	1.71,833E-07	KCNB2	exonic
9	14156633	1.71,833E-07	KCNB2	exonic
9	14149534	1.81,297E-07	KCNB2	exonic
7	28175438	2.01,579E-07	NECTIN1、TRIM29	exonic
18	35159166	2.41,656E-07	RPRM	exonic
30	1471451	2.61,081E-07	AKT3、ZBTB18	exonic
30	1465856	3.81,561E-07	AKT3、ZBTB18	exonic
9	14143658	3.81,589E-07	KCNB2	exonic
30	11987440	3.91,933E-07	DUSP10	exonic
30	2247119	4.01,742E-07	CEP170、A0A9L0SJ43_HORSE	exonic
11	11097567	4.21,223E-07	A0A3Q2L847_HORSE	exonic
23	19616794	4.31,788E-07	TRPM3	exonic
18	39950515	4.41,547E-07	WDSUB1、BAZ2B、TANC1	exonic
1	139529098	4.51,885E-07	LEO1、TMOD3、F6ZPD4_HORSE、MAPK6、GNB5、MYO5C	exonic
11	53463664	4.71,555E-07	PIRT、MYH1、MYH3、SCO1、ADPRM、TMEM220	exonic
1	136344790	4.91,701E-07	NEDD4、PYGO1、PRTG、A0A9L0T9F0_HORSE	exonic
18	43906517	5.01,819E-07	FIGN、ENSECAG00000051747	exonic
9	14144502	5.21,051E-07	KCNB2	exonic
9	10811942	5.61,798E-07	ZFHX4	exonic
18	34719923	5.81,086E-07	A0A9L0S410_HORSE、ARL6IP6、PRPF40A、FMNL2	exonic
9	477090	5.91,913E-07	ENSECAG01,001,041,568;ENSECAG01,001,051,753;ENSECAG01,001,051,863ENSECAG00000059263	ncRNA_exonic

10	53036625	5.91,653E-07	ASCC3	exonic
1	131519687	6.01,372E-07	VPS13C	exonic
7	20612841	6.11,194E-07	POU2AF3、POU2AF1、PPP2R1B、BTG4、HOATZ、LAYN、SIK2	exonic
1	154229808	6.21,114E-07	DPH6	exonic
7	24226205	6.61,247E-07	CADM1	exonic
11	57476256	6.91,711E-07	PMP22、TEKT3、CDRT4	exonic
9	13957740	7.61,883E-07	SBSPON、TERF1、KCNB2	exonic
8	42586062	7.81,435E-07	GATA6	exonic
6	28069884	7.81,777E-07	USP18、TUBA8、PEX26、A0A5F5PYI1_HORSE	exonic
14	15428847	8.31,031E-07	ENSECAG00000028173	ncRNA_exonic
30	12932181	8.61,629E-07	EPRS1、MMGT1、LOC101,051,174、LOC101,051,507、SLC30A10、HHIPL2、A0A9L0SPG0_HORSE、LOC102147783	exonic
30	1466616	9.01,862E-07	AKT3、ZBTB18	exonic
21	15089872	9.71,142E-07	PLK2、GAPT	exonic
1	136343513	9.91,797E-07	NEDD4、PYGO1、PRTG、A0A9L0T9F0_HORSE	exonic

Supplementary Table S5. GWAS analysis results of cannon bone circumference traits in Yanqi horses

**Chromosome**	**Base pair**	**P-value**	**Candidate genes**	**Type**
6	28069889	8.51,322E-09	USP18、TUBA8、PEX26、A0A5F5PYI1_HORSE	exonic
6	28069890	8.51,322E-09	USP18、TUBA8、PEX26、	exonic

			A0A5F5PYI1_HORSE	
14	21933038	1.61,224E-08	SGCD、A0A9L0SHH5_HORSE	exonic
14	22054210	2.21,055E-08	SGCD、A0A9L0SHH5_HORSE、A0A9L0R6T5_HORSE	exonic
18	43907924	3.61,935E-08	FIGN、ENSECAG00000051747	exonic
6	28069884	4.41,618E-08	USP18、TUBA8、PEX26、A0A5F5PYI1_HORSE	exonic
3	117760985	9.21,149E-08	HTRA3、ACOX3、TRMT44、ABLIM2、SH3TC1	exonic
24	5277923	1.51,351E-07	NAA30、CCDC198、SLC35F4	exonic
14	22177301	1.1,465E-07	SGCD、A0A9L0SHH5_HORSE、A0A9L0R6T5_HORSE	exonic
6	28069872	1.81,178E-07	USP18、TUBA8、PEX26、A0A5F5PYI1_HORSE	exonic
6	28069873	1.81,178E-07	USP18、TUBA8、PEX26、A0A5F5PYI1_HORSE	exonic
6	28069875	1.81,178E-07	USP18、TUBA8、PEX26、A0A5F5PYI1_HORSE	exonic
14	28747454	1.91,888E-07	HTR4、FBXO38、LOC101,631,792、A0A3Q2HA16_HORSE、LOC101,631,774、SPINK9、LOC101,781,590、A0A3Q2LLY5_HORSE、LOC101,141,479、A0A9L0R9V3_HORSE	exonic
4	26047728	2.01,678E-07	VOPP1、HGF	intergenic

4	26047729	2.01,678E-07	VOPP1、HGF	intergenic
4	26047737	2.01,678E-07	VOPP1、HGF	intergenic
14	22059252	2.21,384E-07	SGCD、A0A9L0SHH5_HORSE、A0A9L0R6T5_HORSE	exonic
10	702501	2.51,233E-07	ENSECAG01,001,031,696;ENSECAG00000058003	ncRNA_exonic
14	28707532	2.51,251E-07	HTR4、FBXO38、LOC101,631,792、LOC101,631,774、SPINK9、LOC101,781,590、A0A3Q2LLY5_HORSE、LOC101,141,479、A0A9L0R9V3_HORSE	exonic
18	13796012	2.91,164E-07	ENSECAG01,001,041,061;ENSECAG00000030769	intergenic
4	26047722	2.91,513E-07	VOPP1、HGF	intergenic
7	68325571	2.91,336E-07	KCTD21、TENM4、GAB2、USP35、ALG8、KCTD21	exonic
14	28739558	3.11,082E-07	HTR4、FBXO38、LOC101,631,792、A0A3Q2HA16_HORSE、LOC101,631,774、SPINK9、LOC101,781,590、A0A3Q2LLY5_HORSE、LOC101,141,479、A0A9L0R9V3_HORSE	exonic
7	68296720	3.21,358E-07	KCTD21、TENM4、GAB2、USP35、KCTD21	exonic

6	28069864	3.51,785E-07	USP18、TUBA8、PEX26、A0A5F5PYI1_HORSE	exonic
6	28069866	3.51,785E-07	USP18、TUBA8、PEX26、A0A5F5PYI1_HORSE	exonic
14	28750554	3.71,177E-07	HTR4、FBXO38、LOC101,631,792、A0A3Q2HA16_HORSE、LOC101,631,774、SPINK9、LOC101,781,590、A0A3Q2LLY5_HORSE、LOC101,141,479、A0A9L0R9V3_HORSE	exonic
18	16165169	3.81,411E-07	DPP10	exonic
3	93648179	3.81,873E-07	ENSECAG00000059017	ncRNA_exonic
7	24226205	4.01,696E-07	CADM1	exonic
16	69372659	4.01,508E-07	CPNE4	exonic
3	83602269	4.01,705E-07	GABRB1、GABRA4、COX7B2	exonic
14	22057299	4.21,002E-07	SGCD、A0A9L0SHH5_HORSE、A0A9L0R6T5_HORSE	exonic
14	22058443	4.31,184E-07	SGCD、A0A9L0SHH5_HORSE、A0A9L0R6T5_HORSE	exonic
14	22147537	4.41,178E-07	SGCD、A0A9L0SHH5_HORSE、A0A9L0R6T5_HORSE	exonic
14	28607839	5.01,678E-07	HTR4、FBXO38、LOC101,631,792、LOC101,631,774、SPINK9、LOC106781590	exonic

14	28927576	5.61,917E-07	A0A3Q2HA16_HORSE、<br>A0A3Q2I9V8_HORSE、<br>A0A3Q2I2X1_HORSE、<br>LOC101,781,590、<br>A0A3Q2LLY5_HORSE、<br>LOC101,141,479、<br>A0A9L0R9V3_HORSE、<br>LOC106781639	exonic
7	68331440	6.01,946E-07	KCTD21、TENM4、GAB2、USP35、<br>ALG8、KCTD21	exonic
14	21953713	6.01,372E-07	SGCD、A0A9L0SHH5_HORSE	exonic
14	21953698	6.11,531E-07	SGCD、A0A9L0SHH5_HORSE	exonic
9	10811942	6.31,938E-07	ZFHX4	exonic
7	68299955	6.51,396E-07	KCTD21、TENM4、GAB2、USP35、<br>KCTD21	exonic
14	22058586	6.71,485E-07	SGCD、A0A9L0SHH5_HORSE、<br>A0A9L0R6T5_HORSE	exonic
14	21951306	6.91,033E-07	SGCD、A0A9L0SHH5_HORSE	exonic
3	93649402	7.11,629E-07	ENSECAG00000059017	ncRNA_exonic
8	79894344	7.41,538E-07	CCBE1、SEC11C、GRP、LMAN1、<br>RAX	exonic
13	2048093	7.51,453E-07	TTYH3、F6WTT6_HORSE、BRAT1、<br>EIF3B、GNA12、AMZ1、IQCE、<br>SNX8、LFNG、GRIFIN、CHST12	exonic

3	76754764	7.71,951E-07	ENSECAG01,001,051,881;ENSECAG00000038414	intergenic
3	92229818	8.21,329E-07	ENSECAG01,001,031,789;ENSECAG00000046508	ncRNA_exonic
14	22031660	8.61,629E-07	SGCD、A0A9L0SHH5_HORSE、A0A9L0R6T5_HORSE	exonic
28	5263833	8.71,433E-07	ZDHHC17、BBS10、OSBPL8、F6Q085_HORSE	exonic
14	21953985	8.1,957E-07	SGCD、A0A9L0SHH5_HORSE	exonic
9	10198594	8.1,373E-07	ENSECAG01,001,031,727;ENSECAG00000040437	ncRNA_exonic
14	28921042	8.91,218E-07	LOC101,631,792、A0A3Q2HA16_HORSE、A0A3Q2I9V8_HORSE、A0A3Q2I2X1_HORSE、LOC101,781,590、A0A3Q2LLY5_HORSE、LOC101,141,479、A0A9L0R9V3_HORSE、LOC106781639	exonic
18	13758901	8.91,541E-07	ENSECAG01,001,041,061;ENSECAG00000030769	intergenic
14	22069483	9.31,654E-07	SGCD、A0A9L0SHH5_HORSE、A0A9L0R6T5_HORSE	exonic
14	28589248	9.41,688E-07	HTR4、FBXO38、LOC101,631,792、LOC101,631,774、SPINK9、LOC106781590	exonic
19	3085897	9.51,153E-07	ARL14、KPNA4、	exonic

			A0A5F5PFS6_HORSE、A0A9L0RJQ1_HORSE、A0A9L0R7H1_HORSE	
19	3085898	9.51,153E-07	ARL14、KPNA4、A0A5F5PFS6_HORSE、A0A9L0RJQ1_HORSE、A0A9L0R7H1_HORSE	exonic
19	3085900	9.51,153E-07	ARL14、KPNA4、A0A5F5PFS6_HORSE、A0A9L0RJQ1_HORSE、A0A9L0R7H1_HORSE	exonic
14	21949715	9.61,957E-07	SGCD、A0A9L0SHH5_HORSE	exonic
14	21949722	9.61,957E-07	SGCD、A0A9L0SHH5_HORSE	exonic
1	136620211	9.71,306E-07	PIGB、PYGO1、RAB27A、DNAAF4、PRTG、LOC111,761,693、LOC101,781,155、A0A3Q2GXL1_HORSE、LOC101,051,071、A0A9L0RUJ1_HORSE	exonic
9	29276525	9.91,919E-07	XKR4、LOC100053179	exonic
